# Loss of *Sarm1* reduces retinal ganglion cell loss in chronic glaucoma

**DOI:** 10.1186/s40478-024-01736-9

**Published:** 2024-02-08

**Authors:** Huilan Zeng, Jordan E. Mayberry, David Wadkins, Nathan Chen, Daniel W. Summers, Markus H. Kuehn

**Affiliations:** 1 Department of Ophthalmology, The First Affiliated Hospital, Hengyang Medical School, University of South China, Hengyang, Hunan 421001 People’s Republic of China; 2 Department of Ophthalmology and Visual Sciences, The University of Iowa, Iowa City, IA 52242 USA; 3 Iowa City VA Center for the Prevention and Treatment of Visual Loss, Iowa City, IA 52246 USA; 4 Department of Biology, The University of Iowa, Iowa City, IA 52242 USA

**Keywords:** *Sarm1*, SARM1, Glaucoma, RGC loss, Neuroprotection, Axonal degeneration, NAD+ metabolism

## Abstract

Glaucoma is one of the leading causes of irreversible blindness worldwide and vision loss in the disease results from the deterioration of retinal ganglion cells (RGC) and their axons. Metabolic dysfunction of RGC plays a significant role in the onset and progression of the disease in both human patients and rodent models, highlighting the need to better define the mechanisms regulating cellular energy metabolism in glaucoma. This study sought to determine if *Sarm1*, a gene involved in axonal degeneration and NAD+ metabolism, contributes to glaucomatous RGC loss in a mouse model with chronic elevated intraocular pressure (IOP). Our data demonstrate that after 16 weeks of elevated IOP, *Sarm1* knockout (KO) mice retain significantly more RGC than control animals. *Sarm1* KO mice also performed significantly better when compared to control mice during optomotor testing, indicating that visual function is preserved in this group. Our findings also indicate that *Sarm1* KO mice display mild ocular developmental abnormalities, including reduced optic nerve axon diameter and lower visual acuity than controls. Finally, we present data to indicate that SARM1 expression in the optic nerve is most prominently associated with oligodendrocytes. Taken together, these data suggest that attenuating *Sarm1* activity through gene therapy, pharmacologic inhibition, or NAD+ supplementation, may be a novel therapeutic approach for patients with glaucoma.

## Introduction

Glaucoma is a chronic neurodegenerative disease that is characterized by progressive vision loss and changes to the optic nerve head [50]. It affects more than 70 million individuals globally, making it one of the leading causes of irreversible blindness worldwide [50]. High intraocular pressure (IOP) is a main risk factor for the development of the disease and the progression of vision loss, however there are indications that glaucoma is a multi-factorial disease. Factors such as immune responses, mitochondrial dysfunction, and age contribute to the loss of retinal ganglion cells (RGC), and it is likely that their respective contributions to RGC damage vary during glaucoma pathogenesis [40, 50].

A number of recent studies have indicated that metabolic crisis and oxidative stress can cause RGC dysfunction in glaucoma and that cellular NAD+ levels may be a critical determinant of the cells’ survival [21, 28, 36, 40, 44]. Low levels of NAD+ can be caused by several factors, including retinal or systemic metabolic dysfunction, but axonal injury or RGC stress independently cause downregulation of the enzyme NMNAT2, which functions to convert nicotinamide mononucleotide (NMN) to NAD+ [10, 14, 21, 26, 29]. Conversely, providing NAD or its precursors has been shown to attenuate RGC loss in several animal models of glaucoma [43, 51].

Low levels of NAD+ also activate the enzyme sterile alpha and TIR motif-containing 1 (*Sarm1*) that cleaves NAD+ into ADP-ribose, cyclic ADPR, and nicotinamide through dimerization of its TIR domain [9, 42]. Upon SARM1 activation, remaining axonal NAD+ is quickly degraded leading to axonal degradation through Wallerian degeneration and subsequent neuronal death [22, 39, 48]. Furthermore, SARM1 activation can exacerbate axonal degeneration by promoting neuroinflammation through NFκB signaling [19, 25, 30, 54]. Consistent with its role in axonal degradation, studies have shown that inhibition of SARM1 activity can be neuroprotective in rodent models of neurodegenerative conditions, including traumatic brain injury and experimental autoimmune encephalomyelitis (EAE) [2, 4, 13, 23, 31, 34, 45, 54].

The role of SARM1 in injuries affecting ocular neurons is less well established. Deletion of *Sarm1* has been shown to be protective to neurons following optic nerve crush in some studies, but not in others [13, 31]. A protective effect has also been reported following intravitreal injection of TNF-alpha and acute elevation of IOP [25, 31]. However, the chronic nature of glaucoma is a defining characteristic of the disease and a challenge for its management. In this study we sought to determine if loss of *Sarm1* protects RGC morphologically and functionally from glaucomatous damage due to chronic IOP elevation. Our data demonstrate that deletion of the *Sarm1* gene provides significant reduces RGC loss in mice experiencing 16 weeks of moderately elevated IOP. Finally, we demonstrate that naive *Sarm1* knock out (KO) mice display moderate deficiencies in visual acuity, indicating a role of this gene during ocular development.

## Materials and methods

### Animals

Mice used in this study were either normal C57BL/6J (B6) or homozygote C57BL/6J-*Sarm1*^*em1Agsa*^/J (*Sarm1* KO, Jackson Laboratory, stock #034399, Bar Harbor, ME, USA). Mice were housed in the animal facility of the Iowa City Veterans Affair Healthcare System in a 12 h light–dark cycle and were fed chow and water ad libitum. Both male and female mice were used for this study. All studies were approved by the University of Iowa and Iowa City VA Committees for Animal Care and Use and conducted according to the ARVO statement for the use of Animals in Ophthalmology and Vision Research.

### Western blot analysis of endogenous SARM1

Mouse brains from transgenic animals were lysed by dounce homogenization in cold RIPA buffer (50 mM Tris–HCl pH7.4, 1 mM EDTA, 1% Triton X-100, 0.5% sodium deoxycholate, 0.1% SDS, 150 mM NaCl, 1 mM phenylmethylsulfonyl fluoride, and protease inhibitor cocktail from Prometheus). Brain extracts were sonicated for 20 s then centrifuged at 5000×*g* for 5 min at 4 °C. Supernatants were collected and subjected to a second round of centrifugation to remove excess cell debris. The supernatant was mixed with laemmli buffer and 15 µg protein separated by SDS-PAGE. Endogenous Sarm1 and β3-tubulin were detected by western immunoblotting (anti-Sarm1, 1:500, Biolegend # 696602; anti-β3-tubulin, 1:5000, BioLegend #657409). Primary antibodies were detected with a DyLight 680-conjugated goat anti-mouse secondary (1:5000, Invitrogen #35519) and visualized with a LiCor Odyssey Fc imaging system.

### Immunohistochemistry

Optic nerves were dissected immediately following euthanasia and fixed in 4% paraformaldehyde. Nerves were embedded in paraffin and longitudinal sections of 5 micron thickness were prepared. Sections underwent 3 min of heat-induced epitope retrieval (Decloaking Chamber, NxGen) in sodium citrate buffer (10 mM Sodium Citrate, 0.05% Tween 20, pH 6.0). Slides were then blocked with 1% BSA in TBS for 2 h at room temperature and incubated overnight at 4 °C with a 1:100 dilution of the primary antibody (rabbit anti-SARM1 IgG, Cell Signaling #13022, Danvers, MA). Sections were then rinsed and incubated with a 1:1000 dilution of the secondary antibody for two hours (Donkey anti-Rabbit IgG Alexa Fluor® 555, Abcam, Waltham, MA). DAPI was included in all stains to facilitate orientation.

### Induction of elevated IOP

To induce elevated IOP in mice, an adenoviral vector was used that expresses a pathogenic form of human myocilin Ad5RSVmyocilin^Tyr437His^Flag (Ad5myoc, University of Iowa Viral Vector Core, Iowa City, IA, USA), as described previously [18, 53]. Newborn (P2–P5) B6 and *Sarm1* KO mice were subcutaneously injected with Ad5myoc (3 µL of 3 × 10^6^ PfU) to promote tolerance to the vector and limit potential ocular inflammation. At eight weeks of age, mice were anesthetized using 4% isoflurane for induction and 2.5% isoflurane for maintenance with a flow rate of 1L oxygen/min, and 3 × 10^8^ PfU virus in 1 µL of PBS was deposited in the anterior chamber of both eyes via transcorneal injection. 0.5% proparacaine hydrochloride (Bausch + Lomb, Bridgewater, NJ, USA) and 1% tropicamide (Sandoz, Princeton, NJ, USA) eye drops were administered during this process for local anesthesia and dilation. After injection the needle was kept in place for 30 s in order to prevent washout. Both coloration and pulsation of the eye vasculature were monitored throughout the injection to ensure that no ischemic injury occurred. Control mice received an injection of an equivalent amount of sterile PBS.

IOP in anesthetized mice was determined using a rebound tonometer (Tonolab, Colonial Medical Supply, Windham, NH, USA) described previously [24]. IOP measurements were taken between 10 AM and 1 PM by an investigator blinded to the animals’ status.

### Imaging of the optic nerve and measurement of axonal size

Immediately following euthanasia, the optic nerve was removed and fixed in 2% glutaraldehyde and 2% paraformaldehyde. The nerves were then post-fixed in osmium tetraoxide and embedded into Eponate 12 resin. 1 μm thick sections of the optic nerve were taken perpendicular to the length of the nerve. Sections were then stained using 1% p-phenylenediamine (PPD) and images were taken at 100× magnification using a Zeiss Axioscope 5 microscope.

### Measurement of optokinetic reflex (OKR)

Visual acuity in mice was measured using an OptoDrum (StriaTech, Tübingen, Germany). The animal is placed inside on a platform and computer screens surrounding the platform will display a rotating stripe pattern. The reflexive head movements of the mice were recorded by a camera and the system’s software determined if the response of the animal tracked with the pattern it was shown. The spatial frequency of the displayed pattern is either increased or decreased in small increments to determine the level of visual acuity in cycles/degree (c/d) at 99.8% contrast. If the animal is unable to track the rotating stripe pattern by turning their head, it indicates that the pattern is not perceived by the animal and helps to define the level of visual acuity in said animal. Multiple trials are run in each session to ensure the proper analysis of visual acuity by the system. The researcher conducting each test was blinded to the background and status of the mice tested.

### Quantification of RGC density

To analyze RGC density, mice were first euthanized, enucleated, and eyes were fixed in 4% paraformaldehyde for two hours. After fixation, retinas were dissected and placed in 0.3% Triton-X100 in PBS for 6 h. Following three freeze and thaw cycles, retinas were blocked using 1% BSA/0.3% Triton-X100 in PBS for one hour at room temperature. Retinas were then incubated in goat anti-Brn3a primary antibody (Santa Crux, TX, diluted 1:200 in 1% BSA/0.3% Triton-X100/1% DMSO/PBS) at 4 °C for 48 h on a rocker platform. Retinas were washed thoroughly in PBS and binding was visualized after incubation in a donkey anti-goat Alexa Fluor 546 secondary antibody solution (Invitrogen, Carlsbad, CA, USA, 1:200 in PBS) for 3 h at room temperature in the dark. Retinas were again washed in PBS and then whole-mounted using Vectashield (Vector Laboratories, Burlingame, CA, USA). 24 images were taken of each retina at predetermined locations peripherally, mid-peripherally, and centrally at a magnification of 20× using an Olympus BX41 microscope (Olympus, Center Valley, PA, USA). Each image is 330 × 438 μm or 0.1445 mm^2^, thus all 24 images taken together account for 2.89 mm^2^ or approximately 25–30% of the total mouse retinal area. Brn3a+ cells were counted manually by a blinded investigator by using the cell counter plugin for ImageJ software (National Institutes of Health, Bethesda, MD, USA). RGC density per mm^2^ was then calculated by averaging the cell counts of each area of the retina.

### Statistical analysis of data

All calculations were computed using GraphPad Prism 10.0.2 (GraphPad Software, San Diego, CA, USA). *P* values of < 0.01 were considered statistically significant for this study. Comparisons between more than two groups were carried out using ANOVA and Tukey’s multiple comparison test. All data is presented as a mean of animals tested.

## Results

### Cellular localization of *Sarm1* KO in the optic nerve

**Fig. 1:**
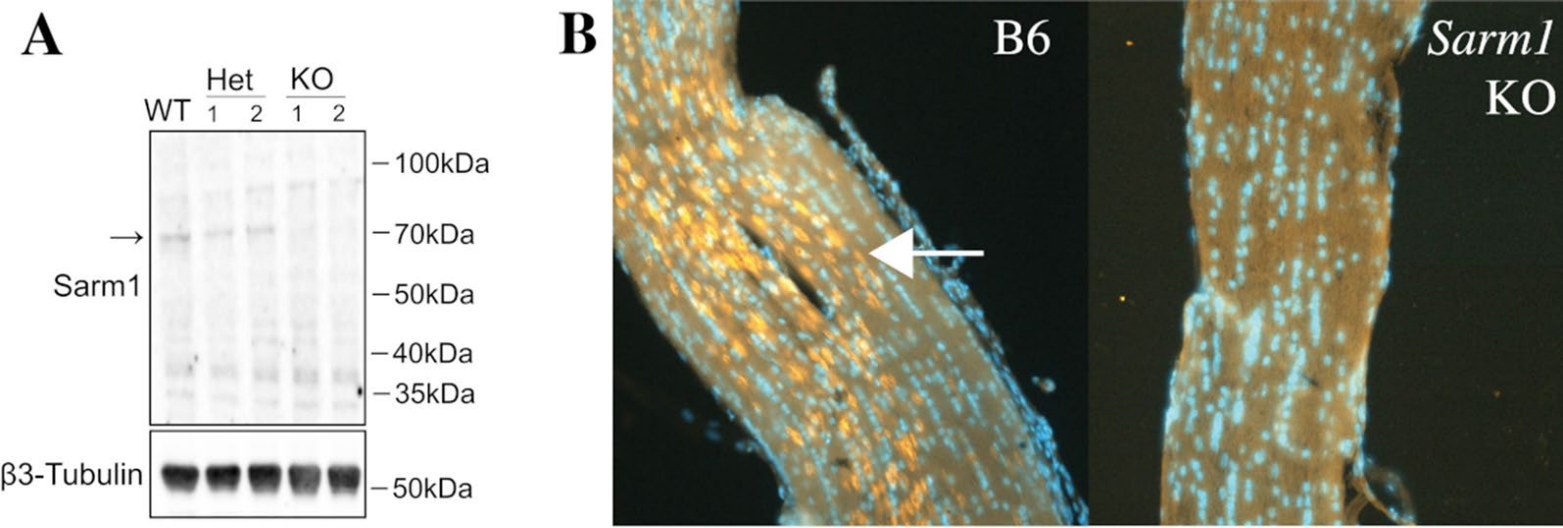
Presence and localization of SARM1 in B6 and *Sarm1* KO mice. **A** Western blot analysis of SARM1 in brain samples of wildtype (WT), heterozygous (het) and homozygous *Sarm1* KO. **B** Immunohistochemical staining of the optic nerve of wild-type (left) and *Sarm1* KO (right) mice with anti-SARM1 antibody. The arrow points to SARM1 + cells

We initially sought to confirm the absence of *Sarm1* in this strain of KO mice and to determine the expression patterns of the protein in the mouse optic nerve and retina. Western blot analysis of brain homogenates from wild-type B6, heterozygote *Sarm1* mice, and *Sarm1* KO mice was carried out to determine if SARM1 protein was detectable in the samples (Fig. 1A). β3 tubulin was used a loading control. Samples obtained from both wild-type B6 and heterozygous *Sarm1* mice reveal a band of approximately 73 kDa, as expected for SARM1, indicating that it is present in these samples. In contrast, this band was not present in brain homogenates of *Sarm1* KO, demonstrating that SARM1 protein is not produced in these mice.

We then used KO and B6 mice to develop immunohistochemistry protocols enabling localization of SARM1 in the mouse optic nerve (Fig. 1B). In longitudinal sections of optic nerve obtained from B6 mice prominent expression of SARM1 is detected throughout the optic nerve, while labeling is completely absent in KO mice. The staining pattern observed is consistent with the size and distribution of oligodendrocytes, rather than axons, suggesting that these cells may be the predominant source of SARM1 in the optic nerve. Under identical conditions, SARM1 immunoreactivity was not apparent in the neural retina, which does not contain oligodendrocytes (data not shown).

### *Sarm1* KO mice display mild ocular developmental defects

**Fig. 2:**
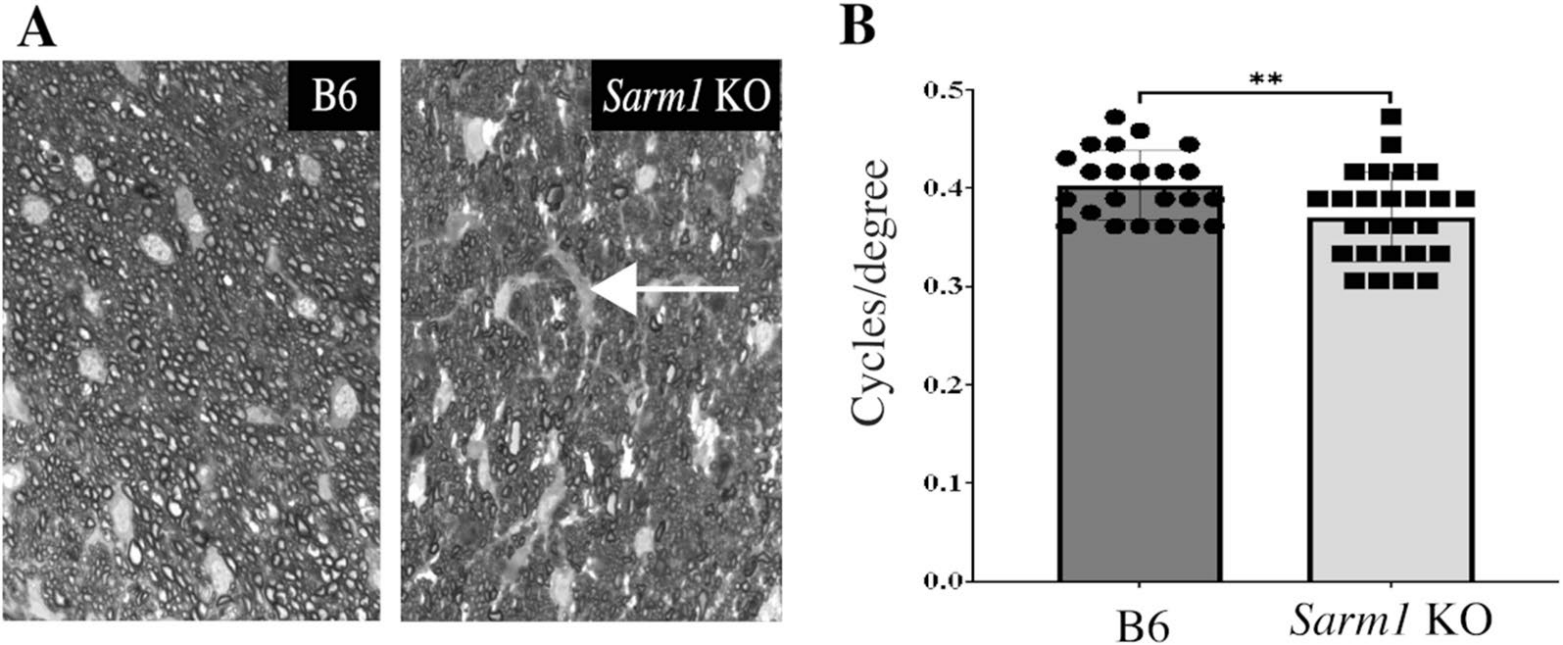
Developmental abnormalities in *Sarm1* KO mice. **A** Cross sections of optic nerves of B6 (left) and *Sarm1* KO (right) mice. While the overall organization of the optic nerve is preserved in KO animals, the overall axonal diameter is reduced and glial elements (arrow) are more voluminous. **B** Naive *Sarm1* KO mice display reduced optokinetic responses when compared to B6 controls. **p < 0.05

In addition to its role during axonal degeneration, SARM1 appears to fulfill a role during embryonic development [5, 48]. Little is known about the impact of SARM1 deletion on ocular structure and function and thus we sought to determine whether *Sarm1* KO mice display developmental defects in the structure of optic nerves or the diameter of optic nerve axons. Toward this goal, optic nerve cross sections were prepared from eight-week old *Sarm1* KO (n = 3) and B6 (n = 3) mice and the density and size of the axons in both groups of animals were examined. Our findings indicate that the overall organization of the optic nerve in *Sarm1* KO is unremarkable when compared to B6. Furthermore, the axonal density in the optic nerves of both groups is almost identical, with an average count of 4347 axons/image in B6 mice, compared to 4400 axons/image in *Sarm1* KO animals (*p* = 0.91, Fig. 2A). However, our data indicate a reduction in the average optic nerve axon diameter in *Sarm1* KO mice when compared to B6. In KO animals, the average major axis measures 0.789 ± 0.1 μm, while the minor axis averages 0.334 ± 0.041 μm. Axon sizes are notably larger in B6 optic nerves, where the major axis averages 1.03 ± 0.044 μm and the minor axis of 0.551 ± 0.03 μm (*p* = 0.0008 and 0.0004, respectively). Although not specifically quantitated here, it appeared that a slightly larger area of the optic nerve was occupied by glial elements in *Sarm1* KO, resulting in similar axonal density despite the reduction in axon caliber.

Subsequent to axonal density analysis, we quantified visual function in B6 (n = 23) and *Sarm1* KO mice (n = 26) using the optokinetic response (OKR) assay, which assesses visual acuity in mice by tracking their responses to visual stimuli. Our data indicate that B6 mice exhibited a mean visual acuity of 0.4028 cycles per degree (Fig. 2B). Conversely, *Sarm1* KO mice demonstrated a marked impairment in visual acuity, with a mean of 0.3707 cycles per degree, a statistically significant reduction (*p* = 0.0084).

### Intracameral injection of Ad5myoc results in chronic IOP elevation

We have previously shown that injections of adenovirus expressing the human myocilin gene with the pathogenic Y437H mutation into the anterior chamber of the eye results in chronic ocular hypertension (OHT) and resultant RGC loss [18, 53]. In order to determine the role of *Sarm1* in glaucomatous RGC loss, Ad5myoc was injected into both eyes of B6 (n = 24) and *Sarm1* KO mice (n = 23). Animals that received adenoviral injection and developed ocular hypertension will be referred to as B6 OHT or *Sarm1* KO OHT for the remainder of the manuscript. Additional B6 (n = 10) and *Sarm1* KO animals (n = 24) were injected with sterile PBS and served as vehicle controls for comparison (B6 control and *Sarm1* KO control, respectively).

**Fig. 3:**
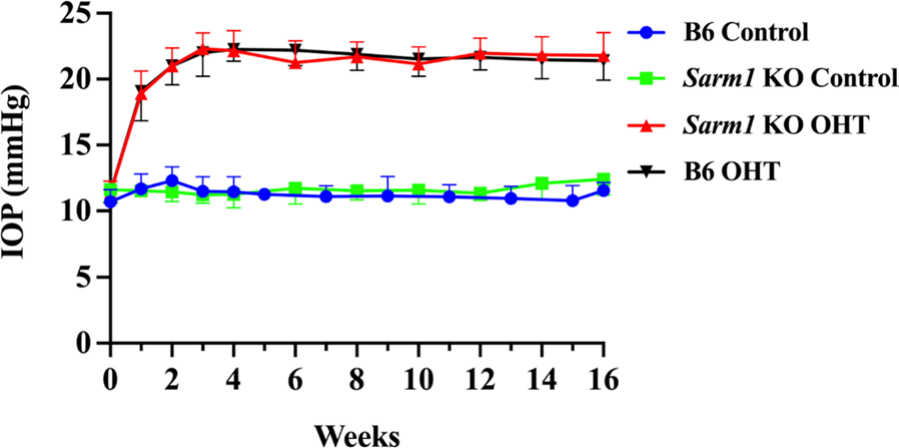
Elevated IOP in B6 mice (black line) and *Sarm1* KO (red line) in mice after injection of Ad5myoc into the anterior chamber of the eye. IOP remains at baseline levels in sham injected B6 (blue line) and *Sarm1* KO (green line). *Note*: error bars denote standard deviation and are only given in either positive or negative direction to avoid overlap on the graph

Following Ad5myoc injection, IOP in both B6 OHT and *Sarm1* KO OHT mice steadily increased, reaching averages of 22.26 mmHg and 22.16 mmHg, respectively, by week four (Fig. 3). For the next 12 weeks, the IOP remained stable in these animals. No differences were observed in the IOP of B6 OHT mice when compared to that of *Sarm1* KO OHT mice. In contrast, neither B6 control nor *Sarm1* KO control animals displayed any elevation of IOP and pressures averaged 11 to 12 mmHg for the duration of the study in both groups.

### Loss of *Sarm1* ameliorates vision loss resulting from chronic high IOP

**Fig. 4:**
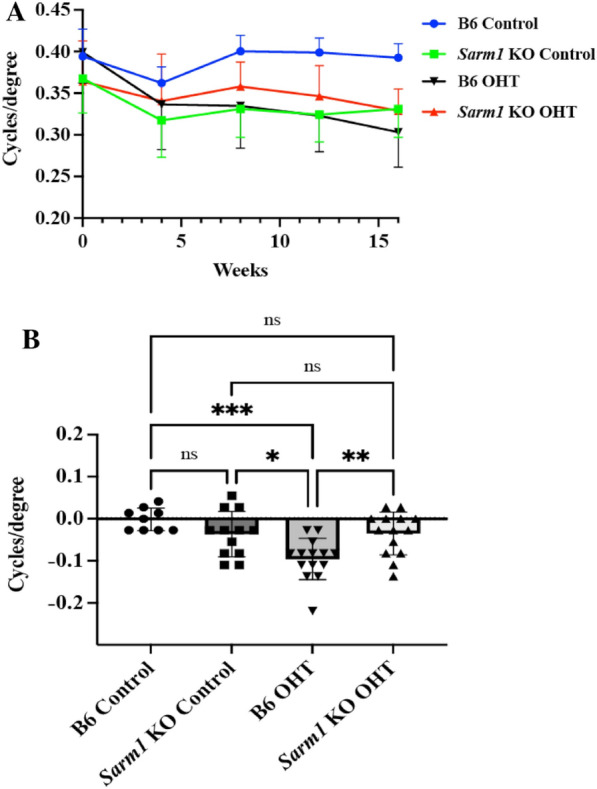
Visual acuity in B6 and *Sarm1* KO mice. **A** OKR responses of B6 controls (blue line), *Sarm1* KO controls (green line), B6 with elevated IOP (black line), and *Sarm1* KO with elevated IOP (red line) mice in cycles/degree. **B** Change in visual acuity from baseline to 16 weeks. *P $\le$ 0.05, **P $\le$ 0.01, ***P $\le$ 0.001, ****P $\le$ 0.0001, and ns = not significant

We then determined the impact of chronic IOP elevation on the visual function in B6 and *Sarm1* KO mice through monthly determination of OKR responses (Fig. 4A). As pointed out above, baseline visual acuity in *Sarm1* KO control is lower than in B6 control and for this reason we defined functional loss as the difference between baseline acuity (week 0) and that measured at the conclusion of the experiment (week 16).

As expected, visual acuity remains stable in the two control groups with normal IOP. B6 control mice (n = 9) lost on average 0.0015 cycles/degree (Fig. 4B). As pointed out above, baseline visual function is lower in *Sarm1* KO (n = 12), but losses in these mice were also negligible in the absence of elevated IOP (0.0365 cycles/degree; *p* = 0.3553 when compared to B6).

In contrast, induction of elevated IOP caused progressive vision loss in B6 mice (n = 14). In this group (B6 OHT), loss of visual acuity averaged 0.0959 cycles/degree during the 16-week period, rendering their visual function significantly worse than that of B6 control animals (*p* = 0.0002). This was not the case in *Sarm1* KO mice with elevated IOP (SARM1 KO OHT; n = 14). These mice exhibited very little loss of visual acuity loss over the course of 16 weeks, despite being exposed to the same level of IOP elevation as the B6 group. Mice in the *Sarm1* KO OHT group lost on average only 0.0352 cycles/degree, which is comparable to changes observed in normotensive *Sarm1* KO control mice (*p* = 0.9999). The data indicates considerable preservation of visual acuity in the *Sarm1* KO mice with elevated IOP when compared to B6 (0.0352 vs. 0.0959 cycles/degree; *p* = 0.0084).

### Loss of *Sarm1* preserves RGC density in eyes with chronic elevated IOP

**Fig. 5:**
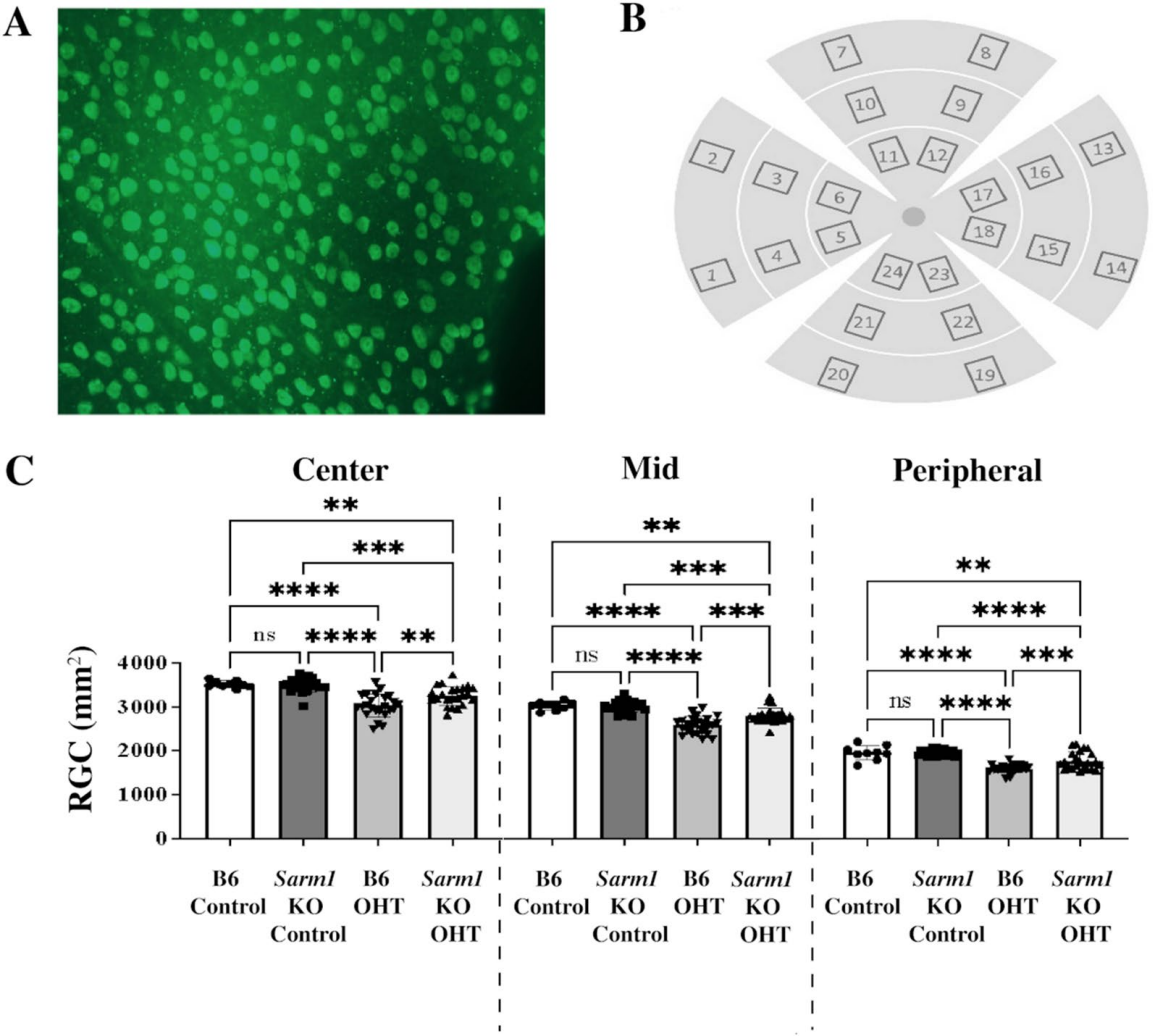
Quantitation of RGC density in eyes of B6 and *Sarm1* KO mice. **A** Whole mounted mouse retina following immunohistochemical staining with RBPMS antibody to indicate RGC (green). **B** Sampling scheme to determine RGC counts in each eye. Data from areas 1, 2, 7, 8, 13, 14, 19, and 20 are averaged to indicate RGC density in the retinal periphery. Images 3, 4, 9, 10, 15, 16, 21, and 22 represent the mid-peripheral area of the retina. Images 5, 6, 11, 13, 17, 18, 23, and 24 represent the central section of the retina. Size of each image equals 330 × 438 μm or 0.1445 mm^2^. **C** RGC density in each area of the retina after 16 weeks of elevated IOP (B6 OHT, *Sarm1* OHT) and in sham injected controls (B6 control, *Sarm1* control). *p $\le$ 0.05, **p $\le$ 0.01, ***p $\le$ 0.001, ****p $\le$ 0.0001, ns = not significant

RGC loss is an important indicator of glaucomatous damage in rodent models of the disease and can be determined following immunohistochemical detection of RGC with antibodies directed against RBPMS (Fig. 5A). Here, we determined RGC density in the peripheral, mid, and central areas of the eyes at the conclusion of the experiment (Fig. 5B, C).

In the central area of the retina, B6 control and *Sarm1* KO control mice with normal IOP display on average 3521 and 3497 RGC per mm^2^, respectively (Fig. 5C and Table 1). The difference between the two groups is not significant (*p* = 0.9957) and agrees with findings presented above indicating similar axon densities in the two groups. As expected, B6 OHT mice displayed a significantly reduced RGC count following 16 weeks of sustained IOP elevation, yielding an average of 3029 RGCs/mm^2^ (*p* < 0.0001). Relative to B6 controls, B6 OHT animals experience a 14% loss of RGC in the central retinal area. *Sarm1* KO OHT mice also display fewer surviving RGC when compared to *Sarm1* KO control animals, averaging 3248 RGC per mm^2^ (*p* = 0.0005). However, RGC loss in *Sarm1* KO OHT mice is only 7.1% when compared to control mice, significantly lower than that observed in B6 (*p* = 0.0027).

**Table 1:** RGC density in each retinal area after 16 weeks

	Center	Mid	Peripheral
B6 Control	3.521 ± 92	3.044 ± 141	1.873 ± 51
B6OHT	3.029 ± 254	2.593 ± 191	1.520 ± 89
Loss	14.0%	14.8%	18.9%
*Sarml* KO Control	3.497 ± 151	3.015 ± 129	1.872 ± 68
*Sarml* KO ORT	3.248 ± 216	2.800 ± 168	1.683 ± 185
Loss	7.1%	7.1%	10.1%
	*p* = 0.00029	*p* = 0.00025	*p* = 0.00328

Values are represented as mean density per mm^2^ $\pm$ SD. Loss is calculated between Controls and OHT. *p* values refer to the differences between B6 OHT and *Sarm1* OHT

Similar results were obtained in the mid area of the retina (Fig. 5C, Table 1). B6 control and *Sarm1* KO control mice display very similar numbers of RGC with averages of 3044 and 3015 RGC/mm^2^, respectively (*p* = 0.9867). RGC density again significantly decreased in B6 OHT animals averaging 2593 RGC per mm^2^, which represents a 14.8% loss when compared to B6 control mice (*p* < 0.0001). *Sarm1* KO OHT mice experience only a 7.1% decrease in RGC (2800 RGC/mm^2^, *p* = 0.0002). These data again indicate substantial RGC protection in *Sarm1* KO OHT when compared to B6 OHT (*p* = 0.0001).

These findings were also observed in the peripheral retina (Fig. 5C, Table 1). Both B6 control and *Sarm1* KO control animals have almost identical numbers of surviving RGC with averages of 1873 and 1872 RGC per mm^2^, respectively (*p* = 0.9995). RGC loss is most pronounced in B6 OHT with an average of 1520 RGC per mm^2^, representing a 18.9% loss in RGC density when compared to B6 controls (*p* < 0.0001). In this area, *Sarm1* KO OHT animals lose 10.1% of RGC when compared to *Sarm1* KO control mice, resulting in 1683 RGC/mm^2^ surviving after 16 weeks of elevated IOP (*p* < 0.0001). This rate of loss is again much lower than in the B6 OHT group (*p* = 0.0002) and indicates that loss of *Sarm1* activity promotes RGC survival in chronic glaucoma associated with elevated IOP.

## Discussion

Therapeutic options for glaucoma are currently limited to reducing IOP, but recent data suggests that modulation of intracellular NAD+ levels can provide neuroprotection in the disease. *Sarm1* is a significant regulator of NAD+ levels, suggesting that it may be a viable target for pharmaceuticals exploration. Since studies in other model systems have indicated a role of SARM1 during embryonic development, our initial efforts were directed to assess the functional and morphologic effects of *Sarm1* ablation on the visual system [5, 48]. During the development of the visual system as much as half of the RGC die after failing to establish connections to the superior colliculus [3]. While it is conceivable that blocking a pathway that contributes to axonal degradation could result in more surviving neurons, we did not observe increased numbers of RGC or optic nerve axons in *Sarm1* KO mice, indicating that this enzyme is not required for this process. We did, however, notice a reduction in the average axon diameter in knockout animals. Smaller axonal size is generally correlated with slower conduction velocity, but experimental verification of this notion was outside the scope of this study [27]. We also noticed reduced optokinetic responses in knockout animals when compared to wild-type mice. Whether this is related to the reduced caliber of optic nerve axons remains to be determined. Axon diameter can influence the timing of signal conduction, whereas the optokinetic response involves a complex integration of visual input, central processing, and motor output making the relationship between axon diameter and the optokinetic response not straightforward [38]. One possible explanation for reduced visual function may be that RGC dendritic arbors in *Sarm1* KO mice appear to exhibit a reduced complexity compared to wildtype counterparts [6]. This could potentially lead to changes in their receptive fields and account for the observed attenuation in optokinetic responses.

Our data further indicate that, within the optic nerve, SARM1 is primarily expressed by oligodendrocytes. Non-neuronal expression of SARM1 has been reported in astroglia [23, 30], and in both cultured and in vivo oligodendrocytes [11]. Using identical experimental procedures, we did not observe SARM1 immunoreactivity in the neural retina, which does not contain oligodendrocytes. Considering the established involvement of SARM1 in axonal degeneration, this finding was unexpected. Within the retina, prior investigations that have either indirectly indicated SARM1 expression by RGCs [31, 49, 52] or have directly demonstrated its presence within retinal homogenates [33]. Furthermore, single cell transcriptomic data from several studies indicate that *Sarm1* is expressed at low levels by RGC and photoreceptor cells [7, 46, 47]. Thus the absence of distinct immunoreactivity in our experiments may be indicative of low Sarm1 levels in RGC, but could also be related to the shortcomings inherent to immunohistochemistry, including epitope availability within the retina.

A main finding of our study is that *Sarm1* ablation confers significant neuroprotection in chronic glaucoma. In our glaucoma model, control mice lose between 19 and 14% of RGC after 4 months of moderately elevated IOP and display a significant decline of visual acuity. In contrast, *Sarm1* KO mice exposed to the same level and duration of IOP elevation, loose only approximately half the number of RGC and maintain visual function similar to baseline levels.

These findings agree with the emerging concept that NAD+ metabolism is a crucial determinant of RGC survival. It is likely that NAD+ levels are decreased in the glaucoma retina and that the activity of the NAD+ synthase NMANT2 decreases in stressed RGC. The physiologic role of SARM1 is to further deplete NAD+ cellular stores to cause rapid degradation and removal of damaged axons. Removal of *Sarm1* most likely confers protection of RGC due to the persistence of higher NAD+ levels due to the absence of the NADase activity of the enzyme [48]. The data further imply that in the absence of SARM1 NAD+ levels in the glaucomatous optic nerve remain sufficiently high to prevent the activation of axon destructive cascades.

Alternatively, it is possible that the observed reduction of RGC in eyes with glaucoma is the result of decreased neuroinflammation in *Sarm1* KO mice. It has been firmly established that retinal inflammation is a significant contributor to RGC damage and axonal loss [40]. *Sarm1* is not only activated by neuroinflammatory signals, but also further promotes inflammation through NF-κB pathway signaling [19, 30, 54]. Deletion of *Sarm1* should lessen NF-κB signaling, resulting in reduced neuroinflammation and enhanced neuronal survival [30, 54].

Although *Sarm1* KO mice clearly experienced less RGC loss than control mice in this glaucoma model, they are not completely protected, underscoring the fact that glaucoma is a multi-factorial disease [50]. Factors impacting the onset and progression of vision loss involve diverse pathomechanisms, including RGC mitochondrial dysfunction and oxidative stress [28, 36], vascular factors [1], and autoimmune processes [17, 32, 35]. Many of these likely persist and will cause damage to RGC even if *Sarm1* disruption prevents the initiation of Wallerian degeneration.

Our data indicating that loss of *Sarm1* affords protection of RGC in glaucoma agree with earlier studies that employed acute injury models to cause RGC death [13, 25, 31]. Importantly, the findings of our research indicate that the protective benefit can be sustained over several months despite unabated IOP elevation, a critical aspect for therapeutic efficacy given the chronic nature of glaucoma [37]. Thus *Sarm1* deletion or inhibition may effectively reduce RGC loss in glaucoma and, given the scarcity of treatments for glaucoma, the potential of *Sarm1* as a therapeutic target is intriguing. Potent inhibitors of *Sarm1* have recently been introduced and may provide viable pharmacologic options [4, 12, 20]. However, chronic systemic suppression of *Sarm1* activity may give rise to undesirable side effects in patients and it is conceivable that gene therapy targeted to the eye may be a more effective strategy [8]. Gene therapy designed to reduce *Sarm1* expression has been successful in some experimental models and may be aided by the fact that even a partial reduction of transcript levels is sufficient to delay axonal degeneration [10, 15, 16, 49].

On the other hand, inhibition of *Sarm1* does not address the metabolic dysfunction that causes its activation and subsequent axon loss. It is possible that supplementing patients with NAD+ or its precursors will restore metabolic balance and prevent activation of *Sarm1* [14, 41, 43, 51]. Alternatively, it may be possible to increase cellular NAD+ levels through gene therapy with NMNAT2, to compensate for its reduced expression levels [10].

Taken together, our data demonstrate that *Sarm1* ablation reduces RGC loss in a rodent glaucoma model with chronic elevated IOP. The profound and sustained effect observed in the model paves the way for further exploration of the potential benefits for the treatment of human glaucoma. Concurrently, our results underscore the potential of NAD+ supplementation as a viable therapeutic strategy to preserve NAD+ homeostasis and prevent *Sarm1*-mediated axonal and RGC degradation [43, 51]. Our data elucidate a pivotal role for *Sarm1* in RGC vulnerability in glaucoma and enhances our comprehension of its long-term effects within a chronic rodent model reflective of human pathology.

## Data Availability

The datasets during and/or analyzed during the current study available from the corresponding author on reasonable request.

## Abbreviations

RGC: Retinal ganglion cells
IOP: Intraocular pressure
OHT: Ocular hypertension
OKR: Optokinetic reflex
EAE: Experimental autoimmune encephalomyelitis
KO: Knockout
NMN: Nicotinamide mononucleotide
